# Association between codon 399 polymorphism in the X-ray repair cross-complementing group 1 gene and risk of prostate cancer in Asians: A study of 4,479 cases and 4,281 controls

**DOI:** 10.12669/pjms.315.7510

**Published:** 2015

**Authors:** Mi Yuanyuan, You Xiaoming, Zhu Lijie, Feng Ninghan

**Affiliations:** 1 Mi Yuanyuan, Department of Urology, Third Affiliated Hospital of Nantong University, Wuxi, China; 2 You Xiaoming, Department of Urology, Third Affiliated Hospital of Nantong University, Wuxi, China; 3 Zhu Lijie, Department of Urology, Third Affiliated Hospital of Nantong University, Wuxi, China; 4 Feng Ninghan, Dept. of Urology, Affiliated WuXi No 2, Hospital of Nanjing Medical University, Nanjing, China

**Keywords:** XRCC1, Codon 399, Polymorphism, Prostate cancer, Meta-analysis

## Abstract

**Objective:**

The polymorphism in codon 399 of the X-ray repair cross-complementing group 1 (XRCC1) gene may subtly alter structure of DNA repair enzymes and modulate the repair capacity. Impaired DNA repair can lead to the development of cancers such as prostate cancer (PCA). Although the association between the *XRCC1 codon 399* polymorphism and PCA risk has been extensively reported, the results have been ambiguous.

**Methods:**

We conducted an updated analysis of 18 case–control studies to determine the association between the *XRCC1 codon 399* polymorphism and PCA risk. We performed a literature search of the PubMed database to identify all eligible articles that reported this association. Odds ratios (ORs) with 95% confidence intervals (CI) were evaluated to assess the association.

**Results:**

Significant associations between PCA risk and *XRCC1 codon 399* polymorphism were found (such as A-allele vs. G-allele: OR = 1.11, 95% CI = 1.01–1.23). Moreover, subgroup analysis based on ethnicity revealed similar significant associations in Asians (such as AA vs. GG: OR = 1.53, 95% CI = 1.19–1.97). Egger’s test did not reveal the presence of a publication bias.

**Conclusions:**

Our updated analysis provides evidence for significant association between *XRCC1 codon 399* polymorphism and PCA risk. Further carefully designed studies should be performed.

## INTRODUCTION

Prostate cancer (PCA) is the most diagnosed cancer and has the second highest mortality in USA.^1^ Moreover, in 2008, a total of 121,797 new PCA cases were diagnosed and 41,996 men died of PCA in the Asia-Pacific region.^2^ PCA is also reported as the most common cancer in elderly men and is the third most commonly encountered malignancy among Pakistani men.^3^,^4^ The cause of PCA is largely unknown, although multiple factors such as exposure to radiation, alcohol consumption, smoking, family history, and diet have been linked with PCA development.^5^ However, not everyone exposed to these risk factors develop PCA, which indicates the differences in individual susceptibility. These differences may be attributed to single-nucleotide polymorphisms in DNA repair genes, which would increase susceptibility to DNA damage from carcinogens.^6^ Genomic stability and integrity are vital for accurate DNA replication. Disruption of DNA sequence integrity can result in gene re-arrangement, translocation, amplification, and deletions, which can in turn contribute to the development of cancers such as PCA.^7^,^8^

The X-ray repair cross-complementing group 1 (XRCC1) gene is located at 19q13.2 and encodes a multi-domain protein that acts as a scaffolding intermediate between ligase III, DNA polymerase-β, and poly-ADP-ribose polymerase.^9^,^10^ XRCC1 can interact with enzymatic components at every stage of DNA strand break repair.^11^ Several polymorphisms have been identified in *XRCC1*. Among these, the *codon 399* polymorphism has been wildly reported to be associated with PCA risk.^12^ This polymorphism is the result of a nucleotide substitution from guanine (Arg) to adenine (Gln) (G to A), and the resulting protein is thought to affect the complex assembly of the base excision repair apparatus or repair efficiency.^13^,^14^

To date, there have been 18 case–control studies in 15 articles^12^-^26^ on the role of the *XRCC1* *codon 399* polymorphism on in the development of PCA. Here, we performed an updated meta-analysis to estimate the association between the *XRCC1* *codon 399* polymorphism and PCA risk.

## METHODS

### Literature search

We tried to include all case–control studies published to date about the association between *XRCC1 codon 399* polymorphism and PCA risk. Eligible studies were found by searching PubMed for relevant reports published between 2002 and 2012. The search terms were “XRCC1” or “X-ray repair cross-complementing group 1,” “polymorphism” or “variant,” and “prostate cancer” or “prostate.” A total of 32 articles were retrieved, of which 15 studies reported on the association between *XRCC1 codon 399* polymorphism and PCA risk.

### Inclusion criteria

(1) association between the *XRCC1 codon 399* polymorphism and PCA risk; (2) case–control study; (3) available genotype frequency; (4) English language; and (5) full-text manuscript.

### Exclusion criteria

(1) no control population; (2) no available genotype frequency; and (3) duplicated studies (we excluded all but the most recent study).

### Data extraction

Data included the following: first author, publication year, country, ethnicity, source of control, each genotype frequency of the case and control groups, genotype methods, and the Hardy–Weinberg equilibrium (HWE) value of the controls.

### Statistical analysis

Odds ratios (ORs) with 95% confidence intervals (CI) were used to measure the strength of the relationship between the *XRCC1* *codon 399* polymorphism and PCA risk. The association between *XRCC1* *codon 399* and PCA risk was determined by 3 different models: allelic contrast (A-allele vs. G-allele), homozygote comparison (AA vs. GG), and the recessive model (AA vs. AG+GG). Subgroup analysis was performed based on the ethnicity and source of case subgroups.

Heterogeneity among the studies was evaluated with a chi-square-based *Q*-test, and the statistical significance of the summary OR was determined with the *Z*-test. Heterogeneity was ruled out when *P >* 0.05 for the *Q*-test; for such studies, the fixed effects model was used, and for other studies, the random effects model was used.^27^,^28^ The HWE was assessed by a chi-square test in controls; *P* < 0.05 was considered significant. Sensitivity analysis was performed on excluded individual studies to assess the stability of the results. Publication bias was assessed by both Egger’s test and Begg’s test.^29^ All statistical tests were performed using the Stata software (version 11.0; StataCorp LP, College Station, TX).

## RESULTS

### Study Inclusion

**Fig.1:**
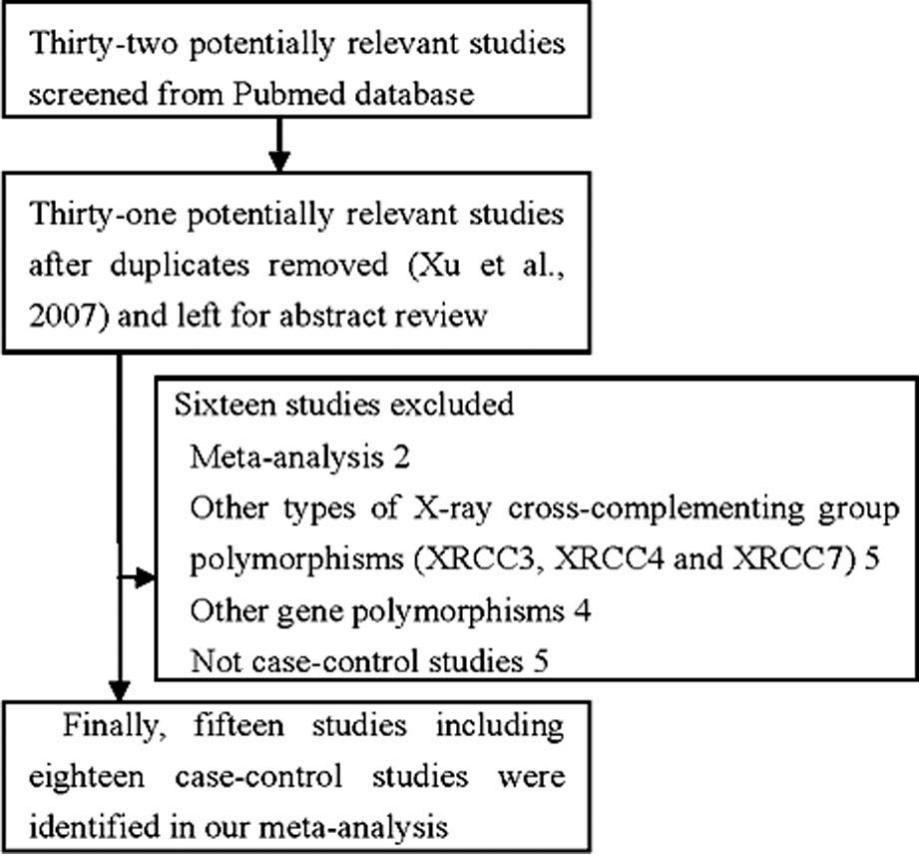
Flowchart illustrating the search strategy used to identify association studies of XRCC1 gene codon 399 polymorphisms and PCA risk for the meta-analysis.

**Fig.2:**
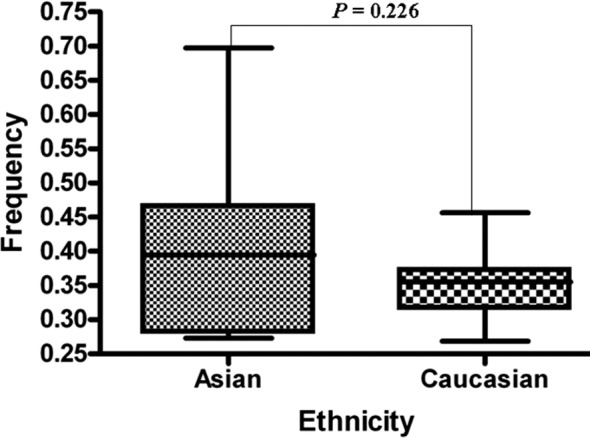
A allele frequencies of XRCC1 gene codon 399 polymorphism among cases stratified by ethnicity (Asian and Caucasian).

**Fig.3:**
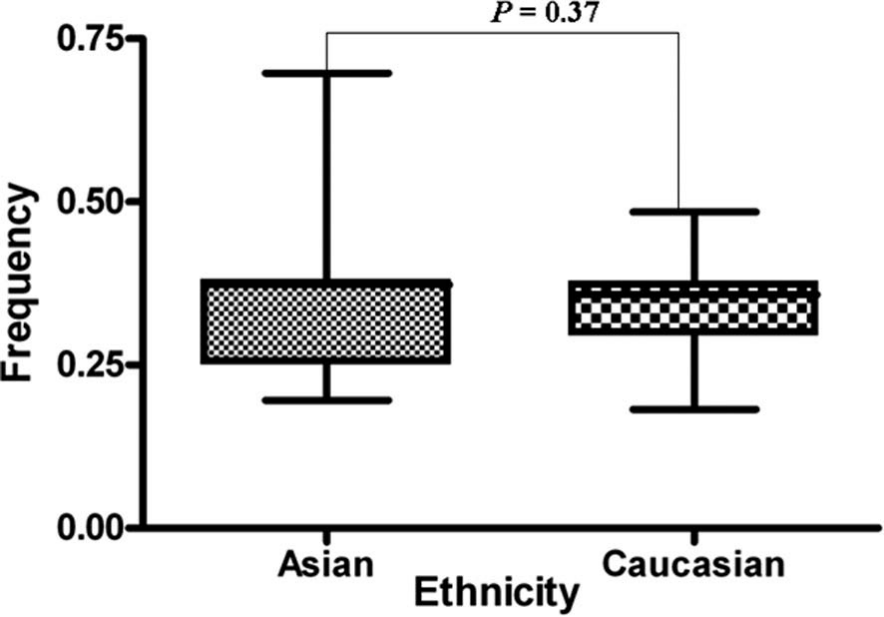
A allele frequencies of XRCC1 gene codon 399 polymorphism among control stratified by ethnicity (Asian and Caucasian).

Of the 32 abstracts retrieved in the PubMed search, 17 did not fulfill the criteria and were excluded. The 15 articles included in the study accounted for 18 case–control studies, which together comprised 4,479 cases and 4,281 controls (Fig.1). Details of the studies are presented in Table-I. Control populations included all study participants with a normal digital rectal examination (DRE) results and serum prostatic specific antigen (PSA) values of < 4 ng/mL. Additionally, they were age-matched and without a personal or family history of cancer. The A-allele % between Asians and Caucasians in the case or control group was >0.05 (Fig. 2 and 3). The distribution of genotypes among controls was in agreement with HWE in all studies except one.^26^

**Table-I:** Study characteristics from published studies on the relationship between condon 399 polymorphisms in XRCC1 gene and prostate cancer.

First author	Year	Country	Etnnicity	Source of control	Cases		Controls				Method	HWE
					AA	AG	GG	AA	AG	GG		
Berhane	2012	India	Asian	HB	40	60	50	24	64	62	PCR–RFLP	0.280
Mittal	2012	India	Asian	HB	49	62	84	43	102	105	ARMS-PCR	0.039
Kuasne	2011	Brazil	Mixed	HB	47	52	73	34	73	65	PCR–RFLP	0.108
Dhillon	2011	Australia	Caucasian	HB	28	49	38	33	60	37	PCR–RFLP	0.386
Mandal	2010	India	Asian	HB	42	51	78	34	83	83	ARMS-PCR	0.098
Gao	2010	USA	Caucasian	PB	56	151	145	10	47	49	PCR-DNS	0.792
Zhang	2010	USA	Caucasian	PB	14	74	102	3	65	127	HTCB-MALD-TOF-MS	0.096
Agalliu	2010	USA	Caucasian	PB	159	576	522	169	590	481	ABI-SNPlex™	0.575
Agalliu	2010	USA	African	PB	4	37	103	2	27	53	ABI-SNPlex™	0.503
Hamano	2008	Japan	Asian	HB	72	54	16	58	50	11	PCR–RFLP	0.962
Hirata	2007	Japan	Asian	HB	15	63	87	10	69	86	PCR–RFLP	0.429
Xu	2007	China	Asian	HB	14	85	108	10	72	153	PCR–RFLP	0.680
Chen	2006	USA	Caucasian	HB	29	104	95	21	87	109	PCR–RFLP	0.552
Chen	2006	USA	African	HB	3	30	90	3	28	84	PCR–RFLP	0.719
Ritchey	2005	USA	Asian	PB	17	53	85	12	99	132	MALDI-TOF-MS	0.226
Rybicki	2004	USA	Caucasian	PB	70	257	245	55	203	179	PCR–RFLP	0.828
Rybicki	2004	USA	Mixed	PB	2	17	46	1	5	37	PCR–RFLP	0.145
van Gils	2002	USA	Caucasian	PB	9	30	37	27	78	77	PCR–RFLP	0.325

PCR-RFLP: polymerase chain reaction and restriction fragment length polymorphism; ARMS-PCR: Amplification refractory mutation specific and PCR; PCR-DNS: PCR and direct nucleotide sequencing; HTCB-MALD-TOF-MS: high-throughput chip-based matrix-assisted laser desorption time-of-flight mass spectrometry; ABI-SNPlex™: Applied Biosystems (ABI) SNPlex™; MALDI-TOF-MS: matrix-assisted laser desorption ionizationtime of flight mass spectrometry; HB: hospital-based; PB: population-based.

### Meta-analysis

**Fig.4:**
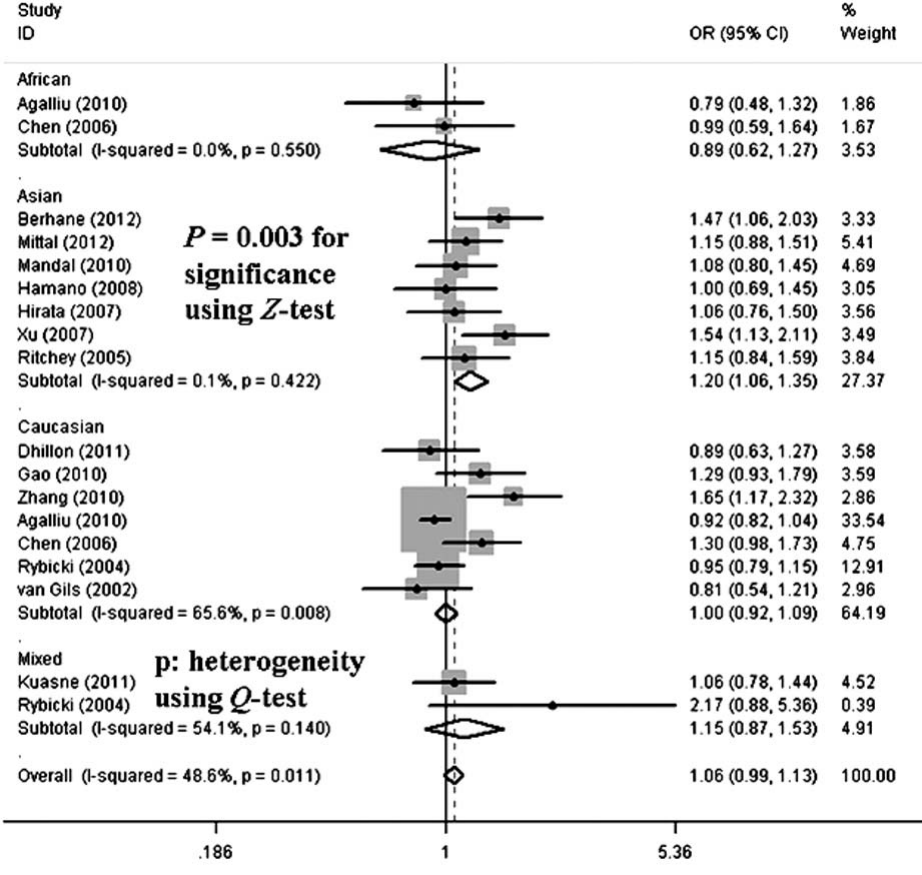
Forest plot of PCA risk associated with the XRCC1 gene codon 399 polymorphism (A-allele vs. G-allele) by ethnicity subgroup. The squares and horizontal lines correspond to the study-specific OR and 95% CI. The area of the squares reflects the weight (inverse of the variance). The diamond represents the summary OR and 95% CI.

**Fig.5:**
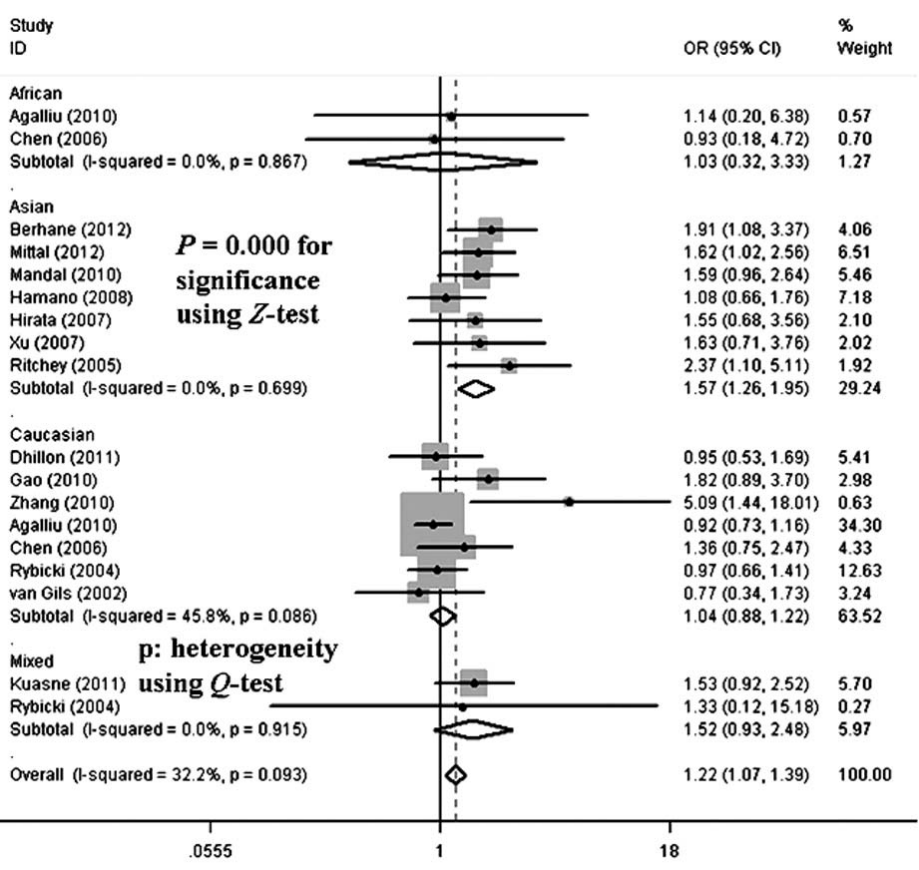
Forest plot of PCA risk associated with the XRCC1 gene codon 399 polymorphism (AA vs. AG+GG) by ethnicity subgroup. The squares and horizontal lines correspond to the study-specific OR and 95% CI. The area of the squares reflects the weight (inverse of the variance). The diamond represents the summary OR and 95% CI.

In total, individuals of the AA genotype or carrying the A-allele had significantly increased risk of developing PCA in all three models (allelic contrast: OR = 1.11, 95% CI = 1.01–1.23, *P* = 0.011 for heterogeneity; homozygote comparison: OR = 1.27, 95% CI = 1.04–1.56, *P* = 0.062 for heterogeneity; the recessive model: OR = 1.31, 95% CI = 1.10–1.57, *P* = 0.093 for heterogeneity) (Table-II). In the subgroup analysis by ethnicity, significant associations were detected in Asian populations but not Caucasians (allelic contrast: OR = 1.20, 95% CI = 1.06–1.35, *P* = 0.422 for heterogeneity, Fig.4; homozygote comparison: OR = 1.53, 95% CI = 1.19–1.97, *P* = 0.639 for heterogeneity; the recessive model: OR = 1.57, 95% CI = 1.26–1.95, *P* = 0.699 for heterogeneity, Fig.5) (Table-II).

**Table-II:** Total and stratified analysis of condon 399 polymorphisms in XRCC1 gene on prostate cancer.

Variables	N^a^	Case/Control	A-allele vs. G-allele		AA vs. GG		AA vs. AG+GG	
			OR(95%CI)	Ph^b^	OR(95%CI)	Ph^b^	OR(95%CI)	Ph^b^
Total	18	4479/4281	1.11(1.01-1.23)	0.011	1.27(1.04-1.56)	0.062	1.31(1.10-1.57)	0.093
*Ethnicity*							
Caucasian	7	2790/2507	1.07(0.91-1.26)	0.008	1.14(0.81-1.61)	0.023	1.12(0.85-1.47)	0.086
Asian	7	1185/1362	1.20(1.06-1.35)	0.422^c^	1.53(1.19-1.97)	0.639^c^	1.57(1.26-1.95)	0.699^c^
Mixed	2	237/215	1.09(0.92-1.30)	0.120^c^	1.51(0.81-1.64)	0.787^c^	1.38(0.94-2.02)	0.971^c^
African	2	267/197	0.90(0.67-1.22)	0.556^c^	0.98(0.31-3.07)	0.936^c^	1.03(0.33-3.24)	0.867^c^
*Source of control*							
HB	10	1668/1753	1.09(1.03-1.16)	0.195^c^	1.19(1.06-1.35)	0.113^c^	1.30(1.13-1.49)	0.589^c^
PB	8	2811/2528	1.07(0.91-1.27)	0.012	1.26(0.85-1.86)	0.023	1.27(0.89-1.81)	0.040

a Number of comparisons.

b P value of Q-test for heterogeneity test.

c Random effects model was used when P value for heterogeneity test <0.10; otherwise, fixed effects model was used.

### Sensitivity analysis and bias diagnosis

**Fig.6:**
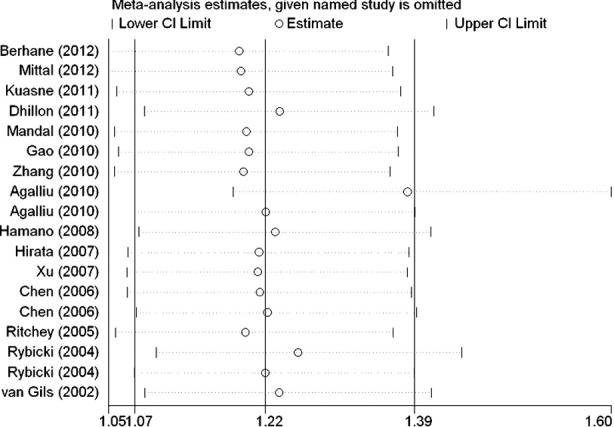
Sensitivity analysis between XRCC1 gene codon 399 polymorphism and prostate cancer risk.

**Fig.7:**
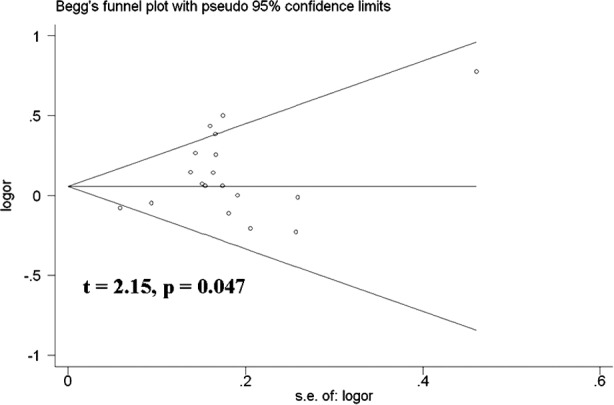
Begg’s funnel plot for publication bias test (A-allele vs. G-allele). Each point represents a separate study for the indicated association. Log [OR], natural logarithm of OR. Horizontal line, mean effect size.

**Fig.8:**
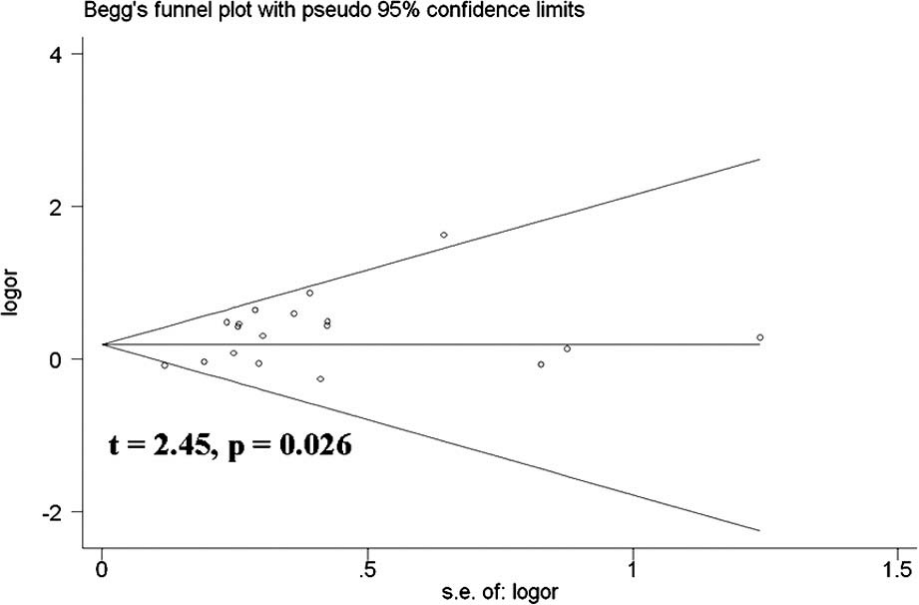
Begg’s funnel plot for publication bias test (AA vs. AG+GG). Each point represents a separate study for the indicated association. Log [OR], natural logarithm of OR. Horizontal line, mean effect size.

Sensitivity analysis was used to determine whether differences in the inclusion criteria of the different studies affect the results. No other single study influenced the summary OR qualitatively (Fig.6). Begg’s test was performed to assess the publication bias of the literature and to provide statistical evidence of funnel plot symmetry. No publication bias was detected (Fig. 7 and 8).

## DISCUSSION

DNA repair systems play an important role in protecting the genome from permanent damage by endogenous and exogenous mutagens, and impairment of these systems has been reported to increase the risk of various types of cancer, including PCA. At least four DNA repair pathways operate on specific types of damaged DNA: base excision repair (BER), nucleotide-excision repair (NER), mismatch repair (MMR), and double-strand break repair.^30^-^33^ *XRCC1* was the first human BER pathway gene to be cloned, and cells lacking this gene product are hypersensitive to ionizing radiation.^34^ XRCC1 works as a stimulator and scaffold protein for other enzymes involved in the BER pathway. Polymorphisms in *XRCC1* that correlate with phenotypic changes have been identified.^35^ One important polymorphism in *XRCC1* is *A399G or R194W*, located in the linker region separating the NH_2_-terminal domain (NTD) from the central BRCT1 (BRCA1 C terminus) domain. This linker region was also suggested to be a potential binding domain for several interacting proteins and is rich in basic amino acids. The substitution of arginine with hydrophobic tryptophan may affect the protein binding efficiency. The present meta-analysis examined 4,479 PCA patients and 4,281 healthy controls to evaluate the association between the *XRCC1* *codon 399* polymorphism and PCA risk.

Our main finding is that the association between the *XRCC1* *codon 399* polymorphism and PCA risk is affected by ethnicity. Significantly strong associations were found between the *XRCC1* *codon 399* polymorphism and PCA in Asians but not in Mixed, Caucasians, or Africans. This suggests that this polymorphism occurs at different frequencies among various ethnic groups and could be considered a biomarker. This difference in distribution could explain the lack of well-replicated results across patient populations of different ethnicities.^36^,^37^ In the future, further studies should compare the distribution of this polymorphism in larger cohorts across various ethnic backgrounds.

### Limitations of the study

Some limitations of our meta-analysis should be mentioned and addressed. First, there were only two Mixed or African case–control studies on *XRCC1* *codon 399* polymorphism and PCA risk. Future studies should focus on these two ethnicities. Second, gene/gene, gene/environment interactions, and even interactions between different polymorphisms, should be included. The stage (TNM and Gleason score) and characteristic (PSA) of PCA should be included if possible. Finally, publication bias was detected, which may influence the power of results.

## CONCLUSION

We provide evidence that *XRCC1* *codon 399* polymorphism could increase PCA risk in Asians. To fully understand the influence of the *XRCC1* *codon 399* polymorphism on susceptibility to PCA, as well as the role of genetic factors in the physiopathology of this disease, further studies in large, standardized, and ethnically diverse populations are needed.
